# Postoperative Hypoglossal Nerve Palsy in Breast Reconstruction Surgery

**DOI:** 10.3390/medicina62050912

**Published:** 2026-05-08

**Authors:** Gil Joon Lee, Woosung Jang, Joon Suk Moon, Byeongju Kang, Jeeyeon Lee, Ho Yong Park, Jeong Yeop Ryu, Kang Young Choi, Jung Dug Yang, Ho Yun Chung, Joon Seok Lee

**Affiliations:** 1 Department of Otorhinolaryngology-Head and Neck Surgery, Sungkyunkwan University School of Medicine, Kangbuk Samsung Hospital, Seoul 03181, Republic of Korea; giljoon.lee@gmail.com; 2 Department of Plastic and Reconstructive Surgery, School of Medicine, Kyungpook National University, Daegu 41944, Republic of Korea; jjh2us@knuh.kr (W.J.); prsryu@knu.ac.kr (J.Y.R.); kychoi@knu.ac.kr (K.Y.C.); lambyang@knu.ac.kr (J.D.Y.); hy-chung@knu.ac.kr (H.Y.C.); 3 Department of Surgery, School of Medicine, Kyungpook National University, Daegu 41944, Republic of Korea; joonsukm@gmail.com (J.S.M.); libertas033@gmail.com (B.K.); j.lee@knu.ac.kr (J.L.); phy123@knu.ac.kr (H.Y.P.)

**Keywords:** hypoglossal nerve, orotracheal intubation, general anesthesia, tongue, patient positioning

## Abstract

*Background/Objectives:* Hypoglossal nerve palsy is a rare but disabling complication of general anesthesia, typically associated with tracheal intubation and head and neck surgery. This study evaluated the incidence, clinical characteristics, and potential mechanisms of postoperative tongue deviation after breast reconstruction and other surgeries performed under general anesthesia with orotracheal intubation. *Methods:* We retrospectively reviewed 240,646 consecutive general anesthetic procedures with orotracheal intubation performed at two tertiary hospitals between September 2011 and October 2025. Eighteen patients who developed new-onset postoperative tongue deviation were identified, and demographic features, surgical department, breast reconstruction status, anesthetic details, patient positioning, laterality of deviation, symptom duration, and recovery outcomes were analyzed. *Results:* Postoperative tongue deviation was documented in 18 patients, corresponding to an overall incidence of approximately 0.01%, most frequently after breast reconstruction (7/18, 38.9%), followed by vascular (27.8%), head and neck tumor (16.7%), neurosurgical (11.1%), and hepatobiliary–pancreatic surgery (5.6%). All seven breast-reconstruction cases occurred at the breast-cancer center hospital, corresponding to 0.31% of 2256 breast reconstructions. The median age was 58.0 years; 66.7% patients were female. Most patients (77.8%) achieved complete recovery, whereas 16.7% had residual deviation. *Conclusions:* Postoperative hypoglossal nerve palsy with tongue deviation is an exceptionally rare event after general anesthesia. In our two-center cohort, it was observed most frequently among patients undergoing breast reconstruction at one participating center; this pattern is confounded by institution-specific anesthetic and positioning practices and should not be interpreted as evidence that the procedure itself carries inherent risk. The findings are hypothesis-generating and suggest that prolonged operating time, repeated intraoperative position changes, and specific head-fixation and tube-fixation practices warrant prospective investigation. Meticulous head–neck alignment, careful tube fixation, and a structured postoperative cranial-nerve check (tongue-protrusion and voice-quality assessment in the recovery room and on postoperative day 1) may aid the early detection of this complication.

## 1. Introduction

The hypoglossal nerve, the twelfth cranial nerve, provides motor innervation to the intrinsic and extrinsic muscles of the tongue as well as selected infrahyoid muscles. Hypoglossal nerve palsy typically presents with ipsilateral tongue deviation, dysarthria, and dysphagia [1,2]. Reported etiologies include intracranial or skull-base tumors, ischemic stroke, head trauma, demyelinating disorders such as multiple sclerosis, Guillain–Barré syndrome, and infectious or inflammatory conditions [1,2]. In the perioperative setting, hypoglossal nerve palsy is rare but has been described following general anesthesia with orotracheal intubation [2,3,4,5,6,7,8,9,10,11,12]. In these cases, external compression or stretching of the extracranial segment of the nerve along the tongue base and mandibular angle is considered the predominant mechanism. Excessive neck flexion or rotation, tight fixation devices, and overinflated tracheal tube cuffs may further increase local pressure, and several studies have linked such malposition—particularly during shoulder surgery—to postoperative tongue deviation [4,7,10,12,13,14,15,16,17]. When hypoglossal palsy occurs together with recurrent laryngeal nerve dysfunction, the clinical constellation is termed Tapia’s syndrome, an uncommon but well-recognized anesthesia-related complication [15,16,17]. Most cases of Tapia’s syndrome are reported after general anesthesia with orotracheal intubation, where compression or stretch neuropathy at the tongue root and adjacent pharyngeal structures has been implicated [15,16,17].

Breast-cancer surgery with immediate reconstruction is anatomically distant from the head and neck, and direct surgical trauma to the hypoglossal nerve is not expected. However, reconstructive procedures often involve prolonged operative times, repeated transitions between supine and semi-sitting or sitting positions, and rigid head fixation to facilitate intraoperative assessment of breast contour and symmetry. Under these conditions, gravitational shift of the head and neck against a fixed tracheal tube may cause unrecognized focal compression or stretch neuropathy along the hypoglossal nerve pathway, even without manipulation near the upper airway [3,4,7,15,16,17]. Despite this plausible mechanism, postoperative hypoglossal nerve palsy after breast reconstruction has been reported only sporadically, with most publications limited to isolated case reports lacking systematic analysis of perioperative risk factors [7,12,13,14,15,16,17].

We conducted this study with an aim to determine the incidence, clinical features, and time course of postoperative hypoglossal nerve palsy presenting as tongue deviation after general anesthesia with orotracheal intubation. We sought to identify surgery- and anesthesia-related factors, including head–neck positioning, airway device fixation, cuff pressure, and operative duration, that may predispose to hypoglossal nerve injury, with particular focus on breast reconstruction cases. By clarifying these patterns, we aimed to provide practical insights to guide anesthesiologists and surgeons in reducing the risk of this rare but functionally significant complication.

## 2. Materials and Methods

The study protocol was approved by the Institutional Review Board of Kyungpook National University Chilgok Hospital (Approval No.: KNUCH 2024-01-008-005), and the requirement for written informed consent was waived because of the retrospective study design. The study was conducted in accordance with the principles of the Declaration of Helsinki.

This retrospective case series study included all patients who developed postoperative tongue deviation after surgery performed under general anesthesia with orotracheal intubation at Kyungpook National University Hospital (KNUH) and Kyungpook National University Chilgok Hospital (KNUCH) between September 2011 and October 2025. During this period, 240,646 general anesthetic procedures with orotracheal intubation were performed at the two institutions; of these, 18 patients developed new-onset tongue deviation after surgery and were identified through review of anesthesia records, postoperative notes, and otolaryngology consultation reports.

For each patient, we collected demographic and anthropometric data (age, sex, height, weight, and body mass index), primary diagnosis, operating department, type of procedure, and treating hospital. Perioperative variables included method of head fixation (shoulder support, headrest, doughnut headrest, or D-shaped frame), type of airway device (rigid or flexible oral tracheal tube, or nasotracheal tube), tube fixation site and depth at the teeth, total operation time, and patient position (supine, lateral decubitus, prone, and use of intermittent sitting or semi-sitting positions). Neurologic outcomes comprised side of tongue deviation (left or right), presence of concomitant hoarseness, duration of symptoms in days, and recovery status at last follow-up, categorized as complete recovery, persistent deviation, or unknown. Symptom duration was defined as the interval from the day of surgery to the visit at which tongue position was first documented as normalized or stable. Comorbidities relevant to peripheral-nerve susceptibility (hypertension, diabetes, dyslipidemia, thyroid disease, and cerebrovascular disease (prior stroke or carotid/intracranial arterial stenosis)) were retrieved where documented. Individual anatomical features (neck length, mandibular prominence, cervical-spine mobility, history of difficult intubation) were not uniformly captured in the retrospective record.

**Figure 1:**
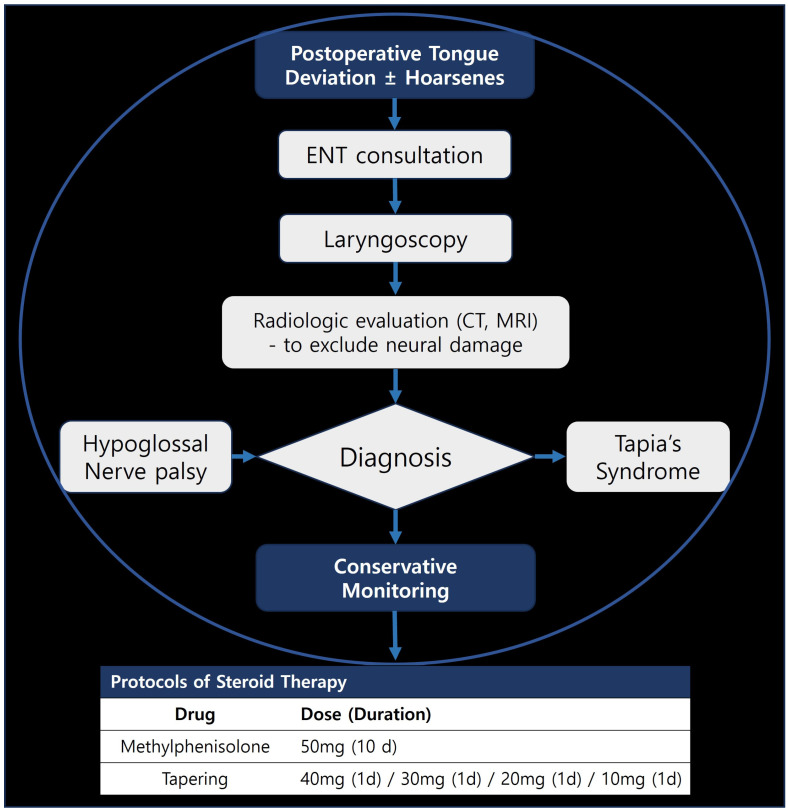
Diagnostic and management algorithm for postoperative tongue deviation with or without hoarseness. The flowchart outlines the stepwise evaluation from bedside detection of tongue deviation to specialist referral, endoscopic airway assessment, and targeted imaging, culminating in classification as isolated hypoglossal nerve palsy or Tapia’s syndrome and guiding subsequent conservative treatment, including an example corticosteroid taper.

At both institutions, patients with suspected upper airway complications after extubation were routinely evaluated by anesthesiologists and, when abnormalities were detected, referred to otolaryngology. In cases of postoperative tongue deviation with or without hoarseness, fiberoptic laryngoscopy was performed to assess vocal cord mobility and exclude structural lesions; computed tomography or magnetic resonance imaging of the head and neck was obtained when central or compressive causes were suspected. If imaging and neurologic examination did not reveal alternative etiologies, a diagnosis of hypoglossal nerve palsy was made; when recurrent laryngeal nerve dysfunction was also present, Tapia’s syndrome was diagnosed. According to institutional practice, patients diagnosed with neural injury received a short course of systemic corticosteroids (e.g., methylprednisolone 50 mg or an equivalent dose of prednisolone for 10 days followed by tapering), and were followed up in the outpatient clinic for at least 6 months whenever feasible. The overall diagnostic and management workflow for patients presenting with postoperative tongue deviation is summarized in a standardized algorithm (Figure 1). Regarding diagnostic workup, all 18 patients underwent formal otolaryngological examination with fiberoptic laryngoscopy and a focused cranial-nerve examination. Neuroimaging (computed tomography and/or magnetic resonance imaging of the head and neck) was obtained in 8 of 18 patients (44.4%) when central or compressive structural causes were clinically suspected (new neurological deficits beyond the tongue, progressive or fluctuating symptoms, or atypical presentation); all eight scans were unremarkable and did not reveal an alternative explanation. In the remaining 10 patients (55.6%), central causes were excluded on clinical grounds in the absence of red-flag neurological features—namely, no other cranial-nerve deficits, no progressive course, no altered mental status, and no focal weakness—consistent with standard otolaryngological practice. The fact that neuroimaging was not performed systematically in every case is acknowledged as a limitation. Regarding corticosteroid treatment: steroids were started between postoperative day 1 and postoperative day 3 once a clinical diagnosis had been made, in the absence of contraindications; the regimen was oral methylprednisolone 50 mg daily (or an equivalent dose of prednisolone) for 10 days, followed by a 5-day taper. This regimen represents historical local institutional practice rather than an evidence-based treatment recommendation. Regarding follow-up completeness: 17 of 18 patients (94.4%) had documented outpatient follow-up for at least one postoperative visit, during which tongue position and function were reassessed, and median follow-up duration exceeded 6 months; one patient (Case #17) was lost to follow-up and is recorded with “unknown” final status. Case identification was performed retrospectively by systematic keyword search of the institutional electronic medical records (including anesthesia records, postoperative nursing notes, surgical operative notes, and otolaryngology consultation reports) for the terms “tongue deviation”, “hypoglossal nerve palsy”, and “Tapia’s syndrome”, applied to all 240,646 general anesthesia procedures performed during the 14-year study period. Potentially eligible records were individually reviewed by a study investigator and included only after formal otolaryngological confirmation of the diagnosis; 18 patients met the inclusion criteria. Because case identification relied on keyword search of clinical documentation, the remaining 240,628 patients (the denominator population) were not individually reviewed for perioperative variables, and a matched case–control dataset was not generated.

Continuous variables are summarized as means with standard deviations or medians with ranges or interquartile ranges, as appropriate, and categorical variables are presented as numbers and percentages. Given the small sample size and descriptive nature of the study, no formal hypothesis testing was performed. As an exploratory, hypothesis-generating step, the incidence of postoperative tongue deviation was compared between breast reconstruction and all other procedures using Fisher’s exact test, and 95% Wilson confidence intervals were calculated for category-specific incidences. Because breast reconstructions were performed exclusively at one of the two participating hospitals, these estimates could not be adjusted for center, surgical team, or standardized intraoperative variables and are interpreted as descriptive associations rather than as adjusted risk estimates. Statistical analyses were performed using SPSS Statistics (version 27.0; IBM Corp., Armonk, NY, USA); a two-sided *p*-value < 0.05 was considered nominally significant for this exploratory analysis.

## 3. Results

During the 14-year study period, 240,646 general anesthetic procedures with orotracheal intubation were performed at KNUH and KNUCH. Clinically recognized postoperative tongue deviation was documented in 18 patients (*n* = 11, KNUCH; *n* = 7, KNUH), corresponding to an incidence of clinically recognized cases of approximately 0.01% (18/240,646) (Table 1). The true incidence—including subclinical or rapidly resolving cases not brought to medical attention—is likely higher, and the reported figure should be interpreted as a lower bound.

**Figure 2:**
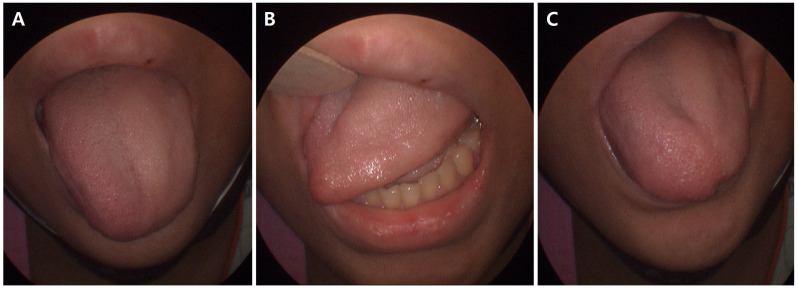
Clinical appearance of left hypoglossal nerve palsy after breast reconstruction. (**A**) Subtle atrophic change on the left side of the tongue with mild leftward deviation at rest. (**B**) Preserved tongue movement when protruding to the right. (**C**) Markedly reduced tongue excursion when attempting to deviate to the left.

The demographic and perioperative characteristics of these patients are summarized in Table 1. The median age was 58.0 years (range, 24–74 years), and 12 patients (66.7%) were female. Median body mass index was 24.2 kg/m^2^ (range, 18.8–28.8 kg/m^2^). The median operation time for surgeries complicated by tongue deviation was 296 min (range, 155–837 min). Operation times tended to be longer at KNUCH than at KNUH (median 355 vs. 213 min, respectively), reflecting the higher proportion of complex reconstructive procedures at KNUCH (Table 1). Clinical presentation included tongue deviation and atrophic changes, as illustrated in Figure 2.

**Figure 3:**
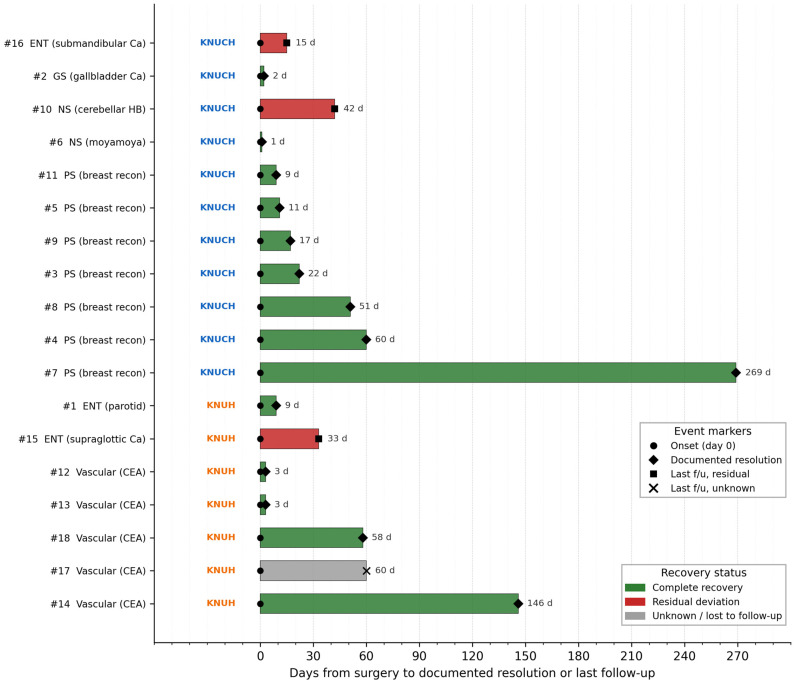
Patient-level timeline of onset and recovery in postoperative tongue deviation (*n* = 18). Each horizontal bar represents one patient. Bar length indicates the interval from the day of surgery (day 0) to documented resolution or last follow-up. Event markers: ● onset of tongue deviation (day 0); ◆ documented resolution; ■ last follow-up with residual deviation; ✕ last follow-up with unknown status (lost to follow-up). Bar color indicates final recovery status: green, complete recovery (*n* = 14); red, residual deviation (*n* = 3); grey, unknown or lost to follow-up (*n* = 1). Patients are grouped by center (KNUCH, Kyungpook National University Chilgok Hospital; KNUH, Kyungpook National University Hospital) and sorted within each center by procedure category and duration to documented resolution. CEA, carotid endarterectomy; Ca, cancer; HB, hemangioblastoma; GS, general surgery; NS, neurosurgery; PS, plastic surgery; ENT, otolaryngology. The figure is descriptive and hypothesis-generating.

The median duration of tongue deviation was 17 days (range, 1–788 days). Fourteen patients (77.8%) experienced complete recovery of tongue position and function, three (16.7%) showed residual deviation at the last follow-up, and one patient (5.6%) was lost to follow-up with unknown final status (Table 2 and Table 3). Complete recovery was observed in 10 of 11 patients (90.9%) at KNUCH and 4 of 7 patients (57.1%) at KNUH, although the small sample size precluded formal statistical comparison. A patient-level timeline of clinical onset and recovery, stratified by center and color-coded by recovery status, is presented in Figure 3.

Clinical characteristics according to hospital are presented in Table 2. Breast reconstruction was the most frequent procedure associated with tongue deviation (7/18, 38.9%), followed by vascular surgery (5/18, 27.8%), head and neck tumor surgery (3/18, 16.7%), neurosurgery (2/18, 11.1%), and hepatobiliary–pancreatic surgery (1/18, 5.6%). All seven breast-reconstruction cases occurred at a single center (KNUCH), so this association is confounded by center and cannot be separated from institution-specific anesthetic and positioning practices. In an exploratory, hypothesis-generating analysis, the center-specific incidence of clinically recognized postoperative tongue deviation after breast reconstruction was 0.31% (95% Wilson CI 0.15–0.64%), and the unadjusted Fisher’s exact odds ratio for breast reconstruction compared with all other procedures across the combined cohort was 67.5 (95% CI 26.1–174.2; *p* < 0.001); because breast reconstructions were performed at only one of the two participating centers, this odds-ratio estimate is fundamentally confounded by center and is interpreted as descriptive rather than as an adjusted risk estimate. For context, the pooled reference rate among non-breast-reconstruction procedures was 11 cases per 238,390 procedures (0.005%); operation-specific denominators for the remaining surgical categories could not be reliably reconstructed from the institutional anesthesia registry without a dedicated manual audit and were therefore not estimated separately. The detailed perioperative profile of the seven affected breast-reconstruction cases is provided in Table 3 (cases #3, #4, #5, #7, #8, #9, and #11); because the 2249 breast-reconstruction patients without recognized tongue deviation were not individually reviewed (see Section 2 for study-design details and the Limitations subsection in Section 4 for the related limitation), a matched case–control comparison of the denominator population was beyond the scope of this retrospective chart-based study. In contrast, cases of tongue deviation at KNUH occurred exclusively owing to vascular and otolaryngology surgery.

Laterality of tongue deviation also differed between hospitals. Overall, 11 patients (61.1%) developed left-sided deviation and 7 (38.9%) developed right-sided deviation. At KNUCH, left-sided deviation predominated (9/11, 81.8%), whereas at KNUH right-sided deviation was more frequent (5/7, 71.4%) (Table 2). All breast reconstruction cases were performed at KNUCH using a doughnut-type headrest with intermittent sitting or semi-sitting elevation, and tongue deviation occurred predominantly on the left side. The individual perioperative profiles of all 18 patients with postoperative tongue deviation have been provided in Table 3.

Across affected patients, the median operation time was 296 min (range 155–837); 13 of 18 procedures (72.2%) exceeded 3 h and 6 (33.3%) exceeded 5 h, identifying prolonged operative duration as the most consistent shared feature of affected cases. Positioning patterns were center- and procedure-specific: doughnut-type headrest with supine–sitting transitions during breast reconstruction at KNUCH (*n* = 7); straight supine with shoulder support during carotid endarterectomy at KNUH (*n* = 5); extended neck positioning during head-and-neck tumor surgery (*n* = 3); rigid head fixation during neurosurgical procedures (*n* = 2); and D-shaped frame during prolonged hepatobiliary surgery (*n* = 1). Documented comorbidities included hypertension (8/18, 44.4%), cerebrovascular disease (prior stroke or carotid/intracranial arterial stenosis) (8/18, 44.4%), diabetes mellitus (5/18, 27.8%), dyslipidemia (3/18, 16.7%), and thyroid disease (2/18, 11.1%); median BMI was 24.2 kg/m^2^. Systematic documentation of individual anatomical features was not available.

## 4. Discussion

We determined the incidence, clinical features, and perioperative risk factors of postoperative hypoglossal nerve palsy presenting as tongue deviation after general anesthesia with orotracheal intubation. Our findings showed that although the overall incidence was extremely rare, occurring in only 18 of 240,646 general anesthetic procedures with orotracheal intubation (0.01%); nevertheless, the incidence was relatively higher in the setting of breast reconstruction, with 7 cases among 2256 reconstructions (0.31%), accounting for more than one-third of all affected procedures. Most patients had favorable outcomes, with complete recovery in 77.8% of patients; however, a subset experienced prolonged symptoms lasting several months or residual deviation at the final follow-up, underscoring the functional impact of this complication.

***Center-specific practice patterns as a major confounder.*** A critical interpretative caveat is that all seven breast-reconstruction cases in our series occurred at a single center (KNUCH). Because breast reconstructions are not performed at KNUH, our dataset does not allow us to separate procedure-related risk from center-specific risk. The higher observed incidence of postoperative tongue deviation in breast reconstruction in this cohort should therefore not be interpreted as evidence that the procedure itself carries inherent risk. Center-specific practice patterns—including the routine use of a doughnut-type headrest, repeated intraoperative transitions to the sitting position, specific tube-fixation technique, local tolerance for prolonged operating times, and local thresholds for otolaryngologic consultation—are major confounders of the observed association, and any one of these factors (alone or in combination) could account for the pattern we report. A multi-center study in which breast reconstruction is performed under varied positioning protocols will be required to disentangle procedure-specific from center-specific effects.

Procedures complicated by postoperative tongue deviation had a longer operation time at KNUCH than at KNUH (median 355 min vs. 213 min); breast reconstructions at KNUCH routinely involved repeated intraoperative transitions between supine and semi-sitting positions with a doughnut-type headrest, conditions that differ qualitatively from the straight supine position with shoulder support used during carotid endarterectomy at KNUH. The cross-category comparison of incidence is unadjusted and confounded by center, so this contextual difference should be interpreted as descriptive rather than as evidence of independent risk.

Previous reports have described postoperative hypoglossal nerve palsy or Tapia’s syndrome mainly after head and neck surgery, neurosurgery, or prolonged intubation in the intensive care unit [3,4,5,6,7,8,9,10,11,14,15,16,17,18,19]. In those settings, both direct surgical trauma and indirect mechanisms such as compression of the nerve at the tongue base, mandibular angle, or transverse process of C1 have been implicated [2,3,4,5,6,7,8,10,11,14,15,16,17]. Our cohort differs in that a substantial proportion of cases occurred after breast reconstruction—a procedure anatomically distant from the hypoglossal nerve—and after carotid endarterectomy or other vascular surgery, where cervical positioning and retraction play a critical role [4,12,14,15,16]. These findings support the view that perioperative positioning and airway management, rather than direct dissection, can be sufficient to precipitate hypoglossal neuropathy under susceptible conditions [2,4,7,14,15,16].

**Figure 4:**
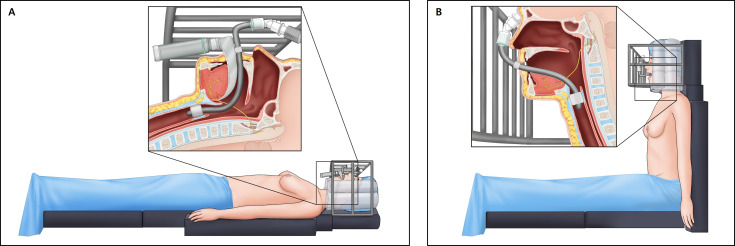
Schematic of general anesthesia and positioning during breast reconstruction (illustrative; hypothesis-generating, not experimental evidence). (**A**) In the supine position, the anesthesiologist performs tracheal intubation using a laryngoscope held in the left hand and advances the tube with the right hand, confirming the appropriate depth before inflating the cuff and securing the tube with tape at the right oral commissure. (**B**) During breast reconstruction, when the patient is transitioned to a sitting position, external supports and drapes exert pressure on the protruding portion of the tube, increasing its curvature within the oral cavity and predisposing the intrapharyngeal segment of the tube to compress the adjacent soft tissues along the hypoglossal nerve pathway.

The pattern of laterality observed in this study is descriptive and should be regarded as speculative. Overall, left-sided tongue deviation was more frequent than right-sided deviation, but the distribution differed between hospitals: left deviation predominated at KNUCH, whereas right deviation was more common at KNUH. All breast-reconstruction cases with tongue deviation occurred at KNUCH, where operations are performed with the head supported in a doughnut headrest and the upper body repeatedly elevated to a semi-sitting position for intraoperative assessment of breast contour (Figure 3). Under these conditions, the weight of the relaxed head and neck may shift asymmetrically against the tracheal tube and surrounding structures, particularly if the tube is fixed at the teeth and the cuff is inflated, and may be associated with focal compression or stretch of the hypoglossal nerve on the dependent side [3,4,7,14,15,16]. Conversely, at KNUH, where tongue deviation was observed predominantly after carotid endarterectomy and other vascular procedures performed with shoulder support in a straight supine position, the direction of nerve injury may be influenced by surgeon- or anesthesiologist-specific preferences for tube fixation and neck rotation (Figure 4). With small per-center numbers, alternative explanations—including chance, different case-mix, and unmeasured differences in tube fixation side, drape tension, or head rotation—cannot be excluded, and any link to anesthesiologist handedness remains a speculative hypothesis that would require a prospective, laterality-matched study to test.

Our intraoperative bench-top simulation using a mannequin and tracheal tube is illustrative only and is not experimental evidence; no control comparison with other long-duration procedures of similar positioning was performed, and no in-vivo pressure or force measurements were obtained. The figure is provided to visualize how elevation of the upper body and subtle changes in head position may accentuate bending of a fixed tube toward one side of the oropharynx (Video S1). In this model, when the trunk is raised to a sitting or semi-sitting posture while the tube is anchored at the teeth and the cuff remains inflated, the cranio-cervical junction effectively shortens relative to the tube length, which may cause the tube to bow laterally and may increase local pressure along the tongue base and adjacent pharyngeal wall [20,21,22,23,24,25,26,27]. Although this setup cannot reproduce in-vivo conditions and was not subjected to controlled measurement, it provides a plausible visualization of how minor differences in fixation and positioning might translate into asymmetric mechanical stress on the hypoglossal nerve; the hypothesis requires prospective testing.

***Candidate risk modifiers for future investigation.*** Four dimensions warrant pre-specified evaluation in prospective studies of this complication. Operation duration: 72% of affected procedures exceeded 3 h, consistent with dose-dependent cumulative exposure of the extracranial hypoglossal segment to low-grade compression or traction. Positioning: configurations differed markedly across surgical categories (doughnut headrest with sitting transitions during breast reconstruction, straight supine during carotid endarterectomy, extended neck during head-and-neck surgery, rigid fixation during neurosurgery), and positioning exposure—rather than the procedure label—is likely the more proximate biomechanical factor. Comorbid background: diabetes, thyroid disease, cerebrovascular disease, and peripheral neuropathy are plausible modifiers of peripheral-nerve susceptibility; a comparator group is required to estimate effect sizes. Individual anatomy: neck length, mandibular prominence, cervical-spine mobility, and airway anatomy were not documented uniformly and could not be analyzed. Future prospective studies should combine standardized preoperative anatomical assessment with continuous cuff-pressure monitoring and videographic documentation of head-and-neck position.

Our results also highlight several modifiable perioperative factors. Tongue deviation occurred after operations with a median duration close to 5 h, and procedures at KNUCH—where most breast reconstructions were performed—were substantially longer than those at KNUH. Prolonged operating times increase the cumulative exposure of the nerve to low-grade compression or traction, particularly when combined with repeated changes between supine and semi-sitting or prone positions [3,4,7,14,15,16]. In addition, although cuff pressure data were not consistently available, overinflation of the tracheal tube cuff and tight fixation at the lips or teeth have been implicated in previous reports of Tapia’s syndrome and isolated hypoglossal palsy [7,14,15,16]. These considerations suggest that meticulous control of cuff pressure, avoidance of excessive neck flexion or rotation, and periodic reassessment of head and tube position after major position changes may help reduce the risk of nerve injury [7,13,14,15,16,23,24,25,26,27].

Most patients in our series received, in addition to observation, a short course of systemic corticosteroids according to historical institutional practice, and the majority recovered fully within several weeks. This regimen should be regarded as historical local practice rather than as an evidence-based treatment recommendation: our series has no untreated comparator group, most cases involved neurapraxia rather than complete transection [3,4,5,6,7,8,9,10,11,12,14,15,16], and spontaneous recovery is well documented in the published literature. The observation that most patients recovered therefore cannot be attributed to corticosteroid therapy, and no statement in this manuscript should be construed as endorsing routine steroid use for this indication; a randomized evaluation would be required to establish whether corticosteroids meaningfully shorten recovery. However, three patients with persistent deviation and at least one patient with very prolonged recovery indicate that even transient hypoglossal palsy can have meaningful consequences for speech, swallowing, and quality of life. Early recognition through routine inspection of tongue movement in the recovery room and during the first postoperative days, followed by prompt otolaryngologic evaluation and imaging when indicated, is therefore essential. This study had some limitations. First, the retrospective design and reliance on clinical documentation likely led to under-recognition of mild or transient cases; therefore, the true incidence may be substantially higher than that reported. Second, the small number of cases precluded formal statistical comparison between subgroups, and the observed differences in laterality or recovery between hospitals should be interpreted as descriptive rather than inferential. Third, detailed information on cuff pressures, exact tube angles, and intraoperative head movements was not systematically recorded, limiting our ability to pinpoint the precise mechanical factors responsible for nerve injury. Fourth, although case ascertainment was performed by retrospective keyword search applied uniformly to both centers, systematic detection bias between the two hospitals is likely: plastic-surgery teams at the center where breast reconstruction was performed (KNUCH) had prior clinical experience with this complication and may have had a lower detection threshold than teams at the other center (KNUH), which means that the apparent higher rate at KNUCH could partly reflect more sensitive detection rather than a truly higher rate of occurrence. In addition, because case identification was performed by retrospective keyword search of the electronic medical record for ‘tongue deviation’, the study design did not generate a matched denominator dataset: the 240,628 patients in whom tongue deviation was not identified were not individually chart-reviewed for perioperative variables, and a direct case–control comparison with the breast-reconstruction denominator (*n* = 2249 without recognized palsy) was therefore not feasible within the present retrospective framework. A prospective case–control or dedicated chart-audit study in the breast-reconstruction population would be required to test whether patient-level or intraoperative variables differ between affected and unaffected patients. Finally, individual-level variables relevant to susceptibility—detailed comorbidity burden, cervical-spine anatomy, neck length and mobility, and baseline cranial-nerve function—were not systematically documented and could not be analyzed; prospective, standardized capture is required for precise characterization of etiopathogenesis.

**Figure 5:**
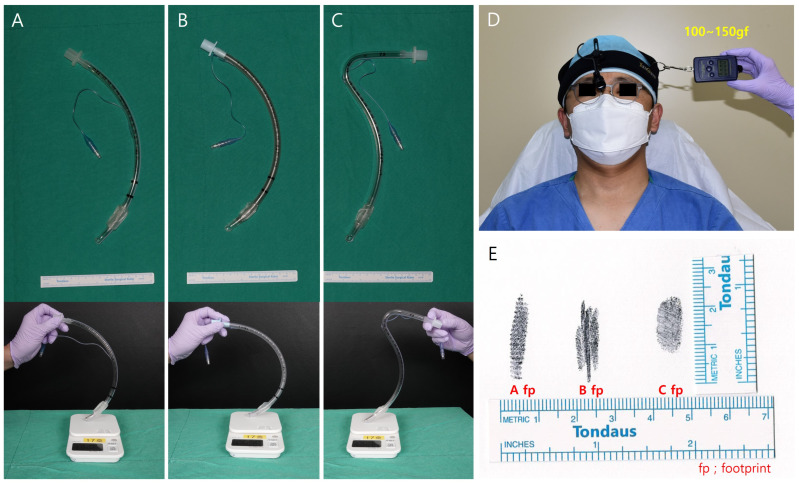
Illustrative bench-top demonstration of tracheal-tube bending characteristics (hypothesis-generating, not experimental evidence). Physical properties of different tracheal tubes and estimated loading in a sitting position. (**A**) Rigid oral tracheal tube showing visible bending when a load of approximately 21–22 g is applied. (**B**) Soft oral tracheal tube demonstrating curvature under a smaller load of approximately 10–11 g. (**C**) A nasal tracheal tube requiring a higher load of roughly 32–33 g before bending is observed. (**D**) In a simulated sitting position with the neck relaxed and tilted to the contralateral side, the dependent side of the head exerts at least 100 g of weight on the supporting surface. (**E**) When the curved segment of each tube is stamped to estimate the contact footprint, the compressed area measures approximately 0.7–0.8 cm^2^ for the rigid tube, 0.9–1.0 cm^2^ for the soft tube, and 0.9 cm^2^ for the nasal tube. The figure is provided to visualize the plausibility of the proposed biomechanical mechanism and should not be interpreted as a validated measurement of in-vivo loading conditions.

***Clinical implications and postoperative cranial-nerve screening.*** Because postoperative hypoglossal nerve palsy has important functional consequences for speech and swallowing, and because its recognition is time-sensitive, we propose that a brief, structured postoperative cranial-nerve check—specifically a 10-s tongue-protrusion and voice-quality assessment performed in the recovery room and again on postoperative day 1—be considered as a routine addition to postoperative care, particularly after prolonged procedures with intraoperative position changes. Prospective documentation of cranial-nerve status at emergence and on postoperative day 1 may also be valuable for medico-legal defensibility. This proposal is offered as a suggestion for prospective evaluation rather than as a validated screening protocol; its impact on incidence, recognition time, and functional outcome would need to be tested formally. Based on the patterns observed in this cohort and on the existing literature, we propose the following practical five-step prevention checklist for prospective evaluation: (i) document tube-fixation site, depth at the teeth, and cuff pressure at intubation; (ii) re-verify head-and-neck alignment and tube position after every transition between supine, semi-sitting, lateral, and prone positions; (iii) avoid prolonged focal pressure from rigid fixation devices, and consider periodic re-positioning of head supports during procedures exceeding 3 h; (iv) perform a brief tongue-protrusion and voice-quality check on emergence from anesthesia and on postoperative day 1; and (v) refer promptly to otolaryngology if new tongue deviation, dysarthria, hoarseness, or dysphagia is detected. Early detection and contemporaneous documentation of cranial-nerve status at emergence and on postoperative day 1 may be valuable both for timely otolaryngological referral and for medico-legal defensibility in the event of an adverse outcome. Despite these limitations, our series represents one of the largest experiences focusing on postoperative hypoglossal nerve palsy in the context of breast reconstruction and other non–head and neck operations. By combining incidence data with detailed perioperative profiles and illustrative imaging and simulation (Figure 2, Figure 4 and Figure 5 and Video S1), we provide practical insights into how positioning techniques, tube fixation, and operative duration may interact to predispose patients to this rare complication. Future prospective studies with standardized monitoring of cuff pressure and head–neck alignment, as well as biomechanical modeling of airway device contact points, may further clarify the pathophysiology and inform targeted preventive strategies [20,21,22,23,24,25]. For peripheral nerves, sustained tissue pressures above critical thresholds have been shown to compromise perfusion and axonal viability [26,27].

## 5. Conclusions

Postoperative hypoglossal nerve palsy with tongue deviation is an exceptionally rare event after general anesthesia. In our two-center cohort it was observed most frequently in patients undergoing breast reconstruction, but all such cases were concentrated at a single participating center, so this observation is confounded by institution-specific anesthetic and positioning practices rather than necessarily reflecting an inherent procedural risk. The findings are hypothesis-generating and are consistent with a possible contribution of prolonged operating time, repeated intraoperative position changes, and specific head-fixation and tube-fixation practices to this complication; however, our retrospective data cannot establish causation, and key biomechanical variables (cuff pressure, head-angle, tube force, repositioning frequency) were not systematically recorded.

Most patients recovered completely with conservative management, whereas a subset experienced prolonged or incomplete recovery, indicating that this complication, although uncommon, is clinically meaningful. Meticulous attention to head–neck alignment, careful control of cuff pressure, reassessment of tube position after posture changes, and routine postoperative inspection of tongue movement may help reduce its occurrence and enable earlier detection.

## Figures and Tables

**Table 1:** Baseline demographics and perioperative characteristics of patients with postoperative tongue deviation.

Characteristic	Total (*n* = 18)	KNUCH (*n* = 11)	KNUH (*n* = 7)
**Sex, *n* (%)**			
Female	12 (66.7)	9 (81.8)	3 (42.9)
Male	6 (33.3)	2 (18.2)	4 (57.1)
**Age, median (range), years**	58.0 (24–74)	48.0 (24–72)	71.0 (51–74)
**BMI, median (range), kg/m^2^**	24.2 (18.8–28.8)	22.9 (18.8–27.2)	25.8 (22.6–28.8)
**Operation time, median (range), min**	296 (155–837)	355 (229–837)	213 (155–313)
**Department, *n* (%)**			
ENT	4 (22.2)	2 (18.2)	2 (28.6)
GS	1 (5.6 )	1 (9.1)	—
NS	2 (11.1)	2 (18.2)	—
PS	6 (33.3)	6 (54.5)	—
Vascular	5 (27.8)	—	5 (71.4)

Values are presented as median (range) for continuous variables and number (percentage) for categorical variables. KNUCH, Kyungpook National University Chilgok Hospital; KNUH, Kyungpook National University Hospital; ENT, ear, nose, and throat; GS, general surgery; NS, neurosurgery; PS, plastic surgery; BMI, body mass index.

**Table 2:** Clinical characteristics of patients with postoperative tongue deviation according to the hospital.

Variable	Total (*n* = 18)	KNUCH (*n* = 11)	KNUH (*n* = 7)
**Female sex, *n* (%)**	12 (66.7)	9 (81.8)	3 (42.9)
**Age, median (IQR), years**	58.0 (41.8–71.0)	44.0 (39.0–58.5)	71.0 (63.5–71.0)
**Height, median (IQR), cm**	161.1 (159.0–165.9)	161.7 (159.6–168.8)	160.0 (158.9–161.5)
**Weight, median (IQR), kg**	62.2 (56.4–68.1)	60.2 (54.6–65.6)	67.0 (62.5–69.8)
**BMI, mean ± SD, kg/m^2^**	23.9 ± 2.6	22.9 ± 2.3	25.5 ± 2.1
**Surgical department, *n* (%)**	PS 7 (38.9); VAS 5 (27.8); ENT 3 (16.7); NS 2 (11.1); GS 1 (5.6)	PS 7 (63.6); NS 2 (18.2); GS 1 (9.1); ENT 1 (9.1)	VAS 5 (71.4); ENT 2 (28.6)
**Breast reconstruction, *n* (%)**	Yes 7 (38.9); No 11 (61.1)	Yes 7 (63.6); No 4 (36.4)	No 7 (100.0)
**Side of tongue deviation, *n* (%)**	Left 11 (61.1); Right 7 (38.9)	Left 9 (81.8); Right 2 (18.2)	Left 2 (28.6); Right 5 (71.4)
**Recovery status, *n* (%)**	Recovered 14 (77.8); Not recovered 4 (22.2)	Recovered 10 (90.9); Not recovered 1 (9.1)	Recovered 4 (57.1); Not recovered 3 (42.9)
**Operation time, median (IQR), min**	279.0 (217.0–352.5)	345.0 (263.0–457.0)	213.0 (190.0–266.0)
Time to recovery, median (IQR), days *	17.0 (9.0–51.0)	17.0 (9.0–46.5)	24.0 (6.0–51.8)

Data are shown as median (interquartile range) for continuous variables and number (percentage) for categorical variables. KNUCH, Kyungpook National University Chilgok Hospital; KNUH, Kyungpook National University Hospital; PS, plastic surgery; VAS, vascular surgery; ENT, ear, nose, and throat; NS, neurosurgery; GS, general surgery; BMI, body mass index; IQR, interquartile range. * Time to recovery was calculated only for patients with available follow-up data; one patient with unknown duration was excluded.

**Table 3:** Individual perioperative profiles of patients with postoperative tongue deviation.

No.	Sex	Age (y)	Surgeon	Diagnosis	Operation	Head Fixation	Op Time (min)	Position	Deviation Side	Recovery (Days)	Status
1	F	72	ENT	Parotid cancer	Radical parotidectomy with simple mastoidectomy	Shoulder support	345	Supine	Left	9	Recovered
2	M	72	GS	Gallbladder cancer	Extended cholecystectomy, liver sectionectomy, colon segmentectomy with Roux-en-Y hepaticojejunostomy	D-shaped frame	837	Supine	Left	2	Recovered
3	F	41	PS	Breast cancer, right	Latissimus dorsi flap breast reconstruction	Doughnut headrest	414	Supine/lateral/prone	Right	22	Recovered
4	F	37	PS	Breast cancer, left	Rotation flap breast reconstruction	Doughnut headrest	190	Supine/prone	Left	60	Recovered
5	F	48	PS	Breast cancer, left	Latissimus dorsi flap with implant breast reconstruction	Doughnut headrest	500	Supine/lateral/prone	Left	11	Recovered
6	F	24	NS	Moyamoya disease	Encephalo-duro-arterio-synangiosis, left	Headrest	258	Supine	Right	1	Recovered
7	F	54	PS	Breast cancer, right	Implant breast reconstruction	Doughnut headrest	268	Supine/prone	Right	269	Recovered
8	F	41	PS	Breast cancer, both	Implant breast reconstruction	Doughnut headrest	355	Supine/prone	Right	51	Recovered
9	F	31	PS	Breast cancer, right	Implant breast reconstruction	Doughnut headrest	303	Supine/prone	Right	17	Recovered
10	M	63	NS	Cerebellar hemangioblastoma	Lateral suboccipital craniotomy and tumor removal	Headrest	687	Lateral decubitus	Right	42	Not recovered
11	F	44	PS	Breast cancer, right	Implant breast reconstruction	Doughnut headrest	229	Supine/prone	Right	9	Recovered
12	F	62	Vascular	Internal carotid artery stenosis	Carotid endarterectomy with patch	Shoulder support	313	Supine	Left	3	Recovered
13	M	71	Vascular	Internal carotid artery stenosis	Carotid endarterectomy with patch	Shoulder support	155	Supine	Left	3	Recovered
14	M	71	Vascular	Internal carotid artery stenosis	Carotid endarterectomy with patch	Shoulder support	213	Supine	Right	146	Recovered
15	M	74	ENT	Supraglottic cancer	Extended modified radical neck dissection, right	Shoulder support	290	Supine	Left	33	Not recovered
16	F	51	ENT	Submandibular gland cancer	Submandibular gland resection with salvage neck dissection, right	Shoulder support	210	Supine	Left	15	Not recovered
17	F	71	Vascular	Internal carotid artery stenosis	Carotid endarterectomy with patch	Shoulder support	242	Supine	Right	NA	Unknown
18	M	65	Vascular	Internal carotid artery stenosis	Carotid endarterectomy with patch	Shoulder support	170	Supine	Left	58	Recovered

ENT, ear, nose, and throat; GS, general surgery; PS, plastic surgery; NS, neurosurgery; Vascular, vascular surgery; NA, not available. BMI was calculated as weight (kg) divided by height (m) squared and rounded to one decimal place.

## Data Availability

All data generated in this study are available within the manuscript.

## Supplementary Materials

The following supporting information can be downloaded at: https://www.mdpi.com/article/10.3390/medicina62050912/s1, Video S1: Animation of breast reconstruction simulation during position change. The clip illustrates (as a hypothesis-generating visualization, not as experimental evidence) how, once tracheal intubation is secured in the supine position, gradual elevation to a semi-sitting or sitting posture may lead to progressive bending of the fixed tube within the oral cavity and oropharynx, which may result in asymmetric contact and possible compression along the presumed course of the hypoglossal nerve.

## Abbreviations

The following abbreviations are used in this manuscript:

**BMI**	body mass index
**ENT**	ear, nose, and throat
**GS**	general surgery
**KNUCH**	Kyungpook National University Chilgok Hospital
**KNUH**	Kyungpook National University Hospital
**NS**	neurosurgery
**PS**	plastic surgery
**VAS**	vascular surgery
